# Supplementary material for: Knowledge on tuberculosis and utilization of DOTS service by tuberculosis patients in Lalitpur District, Nepal
Source: PLoS One. 2021 Jan 25;16(1):e0245686. doi: 10.1371/journal.pone.0245686 (PMC7833137; doi:10.1371/journal.pone.0245686)
Supplement: S1 Data — (ZIP) [file pone.0245686.s001.zip › S1_Data/tools for TB _English_version.pdf]

Questionnaires

Date;--/--/2020

Informed Consent

Title: **Knowledge on Tuberculosis and utilization of DOTS service by Tuberculosis patients in Lalitpur District, Nepal**

Sponsor: None

Introduction of Interviewer

My name is **…..**, final years student of Master of Public Health (MPH) at Novel College of health Sciences, Singamangal, Kathmandu. I am doing this research to assesses the knowledge of TB and utilization of the DOTS service from the DOTS center by TB patients in Lalitpur district, Nepal. Benefit of this research is providing a clear idea of how the tuberculosis patients were living under such circumstance, and how about their (tuberculosis patients) knowledge on TB and how they utilized the DOTS service from public health centers. Additionally, this study helps to provide the better insight in some specific programs are needed for enhancing the knowledge and utilization of DOTS, particularly for those patients whose economic situations extended from low to mid-range.

Respondent's role

You are asking for voluntarily participate in this research. The final decision to be involved in the research is up to you. If you agree, then you are heartly welcome for the information. We will ask to you the questions about your sociodemographic information (age, gender, occupation, education, residence etc), knowledge regarding tuberculosis (do you know TB, Is TB is communicable, Is it treatable etc.), question about quality of life (Are you happy with service provides, happy with your health status, satisfy with treatment modalities etc.). However, if you are not willing to involve in this study then we would appreciate you for listing some words. All the decision will be made base on your (patients) permission. Furthermore, you can ask any questions about this study before you decide to participate. You can ask us to explain any words or information that you may not understand. However, if you do not want the conversation with us, we will not force.

All the collected documents will be store in a safe place at university during the study and destroyed once the data entry is complete. The interview will take about 20-40 minutes.

Confidentiality

The interview will be taken in a private place so that no one can hear what we are saying. We will protect your information and will not write your name in any reports or other publications and presentations. After your agreement and sign on this consent form, we will proceed our study.

Possible Risks

The risk of participating in this study is minimal. Some questions could make you feel uncomfortable. You are free not to answer such questions and to stop the interview at any time you want to do so, without any penalty.

Payment

We will not pay you for your participation.

Leaving the Research

You may leave the research at any time, without hesitation. If you do, it will not change the health services you normally receive.

If you have any questions about this study, please call:

Nirmal Gautam/Rewati Raj Karki,

Chandragiri Ward No-12, Satungol Kathmandu, Nepal

Phone: +977 1 44316317/9851187256

Email: gnirmal655@gmail.com

I understand all the information given above and I agreed to participate in this study by my full interest. And I assure my agreement by my official signature.

Signature: --, Date: -----------/--------/2020

Participant Phone Address if possible: ------------------------------------

Appendix 1. Sociodemographic Variables Questions

Site Name____ Date: ____/____/2020

Study Identification Number(SIDN):		
Dear interviewer, don't forget first to get participant's consent after you tell him/her about the objective and benefits of this study.		
Please ask the participant the following and tick for each question or write on the space provided		
1. Age:	--------------------Years	
2. Gender:	1= Male	☐
	2= Female	☐
3. Distance in average from home of participant to treatment center in Km:	----------------------Km	
4. Education level:	1 = Illiterate	☐
	2 = Informal Education	☐
	3 = 1-5 grade	☐
	4 = 6-8 grade	☐
	5 = 9-12	☐
	6 = Diploma graduate (2 or more years)	☐
	7 = Degree Graduate and above	☐
5. Marital status	1 = Unmarried	☐
	2 = Married	☐

6 Employment condition	1 = labor	☐
	2 = Agriculture	☐
	3 = Private job	☐
	4= Government job	☐
7. Ethnicity	1=JANGATI	☐
	2=BRAMIN	☐
	3= Other	☐
	4=others	☐
8. Family structure	1= Joints	☐
	2= Nuclear	☐
	3=other	☐
9. Income of patients in year	1= less than 100,000 (poor income)	☐
	2= 100,000-500,000 (middle income)	☐
	3= more than 500,000(high income)	☐
10. Residence		
	1= Urban	☐
	2= Rural	☐

2. Question related on knowledge on tuberculosis (TB)

1. Do you know tuberculosis.	1 = Yes	☐
	2 = No	☐ **If No Skip**
2. Tuberculosis is caused by germ/bacteria.	1 = Yes	☐

	2 = No	☐
3. TB can transmit from infected person to uninfected person.	1 = Yes	☐
	2 = No	☐
4. The main TB symptoms are cough ≥ 2 weeks, chest pain, heavy night sweating, appetite loss, weight loss, extreme tiredness or fatigue and coughing up blood with sputum.	1 = Yes	☐
	2 = No	☐
5. TB can be prevented from transmission by, covering mouth and nose during coughing and sneezing, appropriate sputum dropping.	1 = Yes	☐
	2 = No	☐
6. Is defaulter/relapse TB is cured	1 = Yes	☐
	2 = No	☐
**7.** Can tuberculosis be prevented by not sharing the utensils with an infected person	1 = Yes	☐
	2 = No	☐
8. There is effective medical treatment for TB disease.	1 = Yes	☐
	2 = No	☐
9. How long is the treatment for tuberculosis (not MDR or XDR TB)	1 = 2 months	☐
	2 = 8 months	☐
	3 =8 to 12 months	☐
	4= 12 to 18 months	☐

Utilization of the Directly-Observed Therapy, Short Course (DOTS)

1. Do you know about Directly-Observed Therapy, Short Course (DOTS)?		
	1 = Yes	☐
	2 = No	☐
2. Did you regular and complete utilized the DOTS service in first 2 month (initial phase)?		
	1 = Yes	☐
	2 = No	☐
3. Why you did not utilize the regular and complete the initial phase of the DOTS service,		
	1= long distance	☐
	2=lack of transportation	☐
	3=physical weakness	☐
	4=Others	☐
4. Did you regular and complete utilized the DOTS service in last 6 months period (initial phase and continuous phase)?		
	1= Yes	☐
	2= No	☐
5. Why you did not utilize the continuous phase of DOTS service	1= long distance<br>2= lack of transportation<br>3= physical weakness<br>4= Others	☐<br>☐<br><br>☐
