# Supplementary material for: Knowledge on tuberculosis and utilization of DOTS service by tuberculosis patients in Lalitpur District, Nepal
Source: PLoS One. 2021 Jan 25;16(1):e0245686. doi: 10.1371/journal.pone.0245686 (PMC7833137; doi:10.1371/journal.pone.0245686)
Supplement: S1 Data — (ZIP) [file pone.0245686.s001.zip › S1_Data/Tool_Nepali_version.pdf]

सहमति फारम

नमस्ते, मेरो नाम ……………………………………, व्यावसायिक रूपमा म जनस्वास्थ्य कार्यकर्ता हो । मेरा अनुसन्धान शीर्षक **"ललितपुर जिल्ला बसोबस गर्न क्षयरोग बिरामी को क्षयरोग सम्बन्धी ज्ञान र डटस सर्भिस उपयोग"** पहिचान गर्नको लागि अनुसन्धानको डाटा संकलन गर्दैछु । अब, म तपाईलाई, तपाईंको सम्पूर्ण चासोको साथ यस अनुसन्धानमा भाग लिन तपाईंको इच्छुकता सोध्न चाहन्छु। यो अनुसन्धान क्षयरोगको उपचार बढाउन र क्षयरोग प्रतीको अविश्वास रोक्न तपाई र तपाईंको समुदायका लागि महत्त्वपूर्ण छ । साथै, म तपाईलाई यो कुरा बताउन चाहन्छु कि यस अध्ययनमा भाग लिने वा नलिने तपाईसँग पूर्ण अधिकार छ र अध्ययन अवधिको प्रक्रियामा तपाईसँग कुनै पनि समयमा सहभागिता रोक्ने अधिकार छ । साथै तपाईले दिनुभएको जानकारी गोप्य रहनेछ र यो अध्ययनको लागि मात्र प्रयोग गरिनेछ।

प्रिय सहभागी, तपाइँको सहयोग को लागी धन्यवाद र अब म तपाईंकोलागी यो लिखित सहमति फारम पढ्छु वा तपाई पढ्न सक्नुहुन्छ र तपाई यस अनुसन्धानको सबै उदेश्य , प्रकृया,सुविधाहरू र अधिकारहरू बुझेर यस अध्ययनमा तपाईंको पूर्ण इच्छासहित भाग लिने कुरामा सहमत भई , तपाई मेरो लागि हस्ताक्षर गर्नसक्नुहुनेछ ।

म माथि दिइएका सबै जानकारीहरू बुझ्दछु र म मेरो पूर्ण चासो लिएर यस अध्ययनमा भाग लिन सहमत छु। र म मेरो आधिकारिक हस्ताक्षर द्वारा मेरो सम्झौता लाई आश्वस्त पार्छु।

हस्ताक्षर : ---,

मिति -----------/--------/20--

सहभागीको फोन, ठेगाना (यदि सम्भव भएमा): ------------------------------------

<u>प्रश्नावलीहरू</u>

१. सामाजिक जनसांख्यिकीय

स्थान नाम.. मिति: ---------/--------/20—

प्रिय साक्षात्कारकर्ता,यस अध्ययनको उद्देश्य र फाइदाहरूको बारेमा सहभागीलाइ बताउनु भए पछि सहभागीको सहमति लिन नबिर्सनुहोस् ।		
कृपया सहभागीलाई निम्न प्रश्नहरू सोध्नुहोस् र चिन्ह √ लगाउनुहोस् वा प्रदान गरिएको ठाउँमा लेख्नुहोस ।		
१. उमेर (पुरा भएको)	--------------------	
२. लिंग	१ = पुरुष	☐
	२= महिला	☐
३. सहभागी को घर बाट स्वास्थ्य संस्थाको दुरी कि मी	---------------------- कि मी	
४. शैक्षिक तह	१ = अशिक्षित	☐
	२= शिक्षित (अनौपचारिक)	☐
	३= १- ५ कक्षा	☐
	४= ६ -८ कक्षा	☐
	५= ९-१२ (तयारी पूर्ण)	☐
	६= माध्यमिक (९ देखि १२)	☐
	७= डिग्री स्नातक वा माथि	☐

५. वैवाहिक स्थिति	१ = अविवाहित	☐
	२= विवाहित	☐
६. रोजगारको अवस्था	१ = श्रम	☐
	२= कृषि	☐
	३= निजी जागीर	☐
	४= सरकारी जागीर	☐
७. बासस्थान	१ = शहरी	☐
	२= ग्रामिण	☐
८. जातीयता	१ = जनजती	☐
	२= बाहुन	☐
	३= अन्य	☐
९.परिवारको किसिम	१ = सन्युक्त	☐
	२= एकल	☐
	३= अन्य	☐
१०. आय स्थिति		
	१ = १००,००० भन्दा कम् (गरीब)	☐
	२= १००,००० - ५००,००० सम्म (मध्यम)	☐
	३= ५००,००० भन्दा धरै (धनी)	☐

२. क्षयरोग ज्ञान सम्बन्धित प्रश्नावलीहरु

१. तपाईं लाई क्षयरोग (टि वी)भनेको थाहा छ।	१ = छ	☐
	२= छैन	☐ **छैन भने स्किप गर्नुस्**
२. के टि वी जीवाणु र ब्याक्टेरियाबाट हुन्छ	१ = सहि	☐
	२= गलत	☐
३. के टि वी सन्क्रमित व्यक्तिबाट स्वस्थ्य व्यक्तिलाई सर्छ।	१ = सहि	☐
	२= गलत	☐
४ के टि वी सन्क्रमित व्यक्ति ले खाने भाँडा बाँडफाँड रोकथाम बाट, टिवी सर्न बाट कम् गर्न सकिन्छ	१ = सहि	☐
	२ = गलत	☐
५. खोकी र हाछियौ समयमा मुख, नाक छोप्दा, जथाभावी नथुक्दा, र खकार लाई उचित बेवस्तपान गर्दा टि वी रोगलाई फैलिन बाट रोकथाम गर्न सकिन्छ।	१ = सहि	☐
	२= गलत	☐

६. के DEFULTER /RELAPSE टिबी निको हुन्छ।	१ = सहि	☐
	२ = गलत	☐
७. टि वी का मुख्य लक्ष्यणहरु दुई हफ्ता भन्दा बढी खोकी लाग्नु, छाती दुस्खनु ,राती धेरै पसिना आउनु ,भोक नलाग्नु ,तौल घट्नु , धेरै थकाई लाग्नु र खकारमा रगत देखिनु हो ।	१ = सहि	☐
	२= गलत	☐
८.टि.बी रोग प्रभावकारी उपचार बाट निको हुन्छ।	१ = सहि	☐
	२= गलत	☐
९.. टीबी उपचारको लागि कुल कती समय नयाँ बिरामीको लागि लाग्छ (MDR XDR र RESISTANCE /DEFAULTER बाह्यक)।	१ = २ महिना	☐
	२= ८ महिना	☐
	३= ८ देखी १२ महिना	☐
	४= १२ देखी १८ महिना	☐

डटस सर्भिस उपयोग सम्बन्धित प्रश्नावलीहरु

१ . तपाईं लाई डटस सर्भिस भनको थाहा छ	१. = छ	☐
	२ =. छैन	☐
२ के तपाईं ले सुरुको २ महिना डटस सर्भिस नियमित उपयोग गर्नु भयो	१. = नियमित उपयोग	☐
	२.= नियमित उपयोग छैन	☐
३. यदी डटस सर्भिस नियमित उपयोग गर्नु भएन भन्न्य किन गर्नु भएन	१ =. लामो बाटो को करण ले	☐
	२. =यातायात को करण ले	☐
	३.= कम्जोर को करण ले	☐
	४. = अन्न्य	☐
४. तपाईं ले अन्तिम को छ महिना डटस सर्भिस नियमित उपयोग गर्नु भयो	१. = नियमित उपयोग	☐
	२ =. नियमित उपयोग छैन	☐
५ यदी डटस सर्भिस नियमित उपयोग गर्नु भएन भन्न्य किन गर्नु भएन	१. = बाटो को करण ले	☐
	२ =.यातायात को करण ले	☐
	३. = कम्जोर को करण ले	☐
	४. = अन्न्य	☐
