# Supplementary material for: Knowledge on tuberculosis and utilization of DOTS service by tuberculosis patients in Lalitpur District, Nepal
Source: PLoS One. 2021 Jan 25;16(1):e0245686. doi: 10.1371/journal.pone.0245686 (PMC7833137; doi:10.1371/journal.pone.0245686)
Supplement: Attachment — Submitted filename: renamed_abc7b.docx [file pone.0245686.s002.docx]

Responses to Editor’s and Reviewer’s Comments

| **Editor Comments** | **Answer** |
| --- | --- |
| 1. Please ensure that your manuscript meets PLOS ONE's style requirements, including those for file naming. The PLOS ONE style templates can be found at. | Thank you for this information, we have ensured that the PLOS ONE’s style requirement is maintained in revised manuscript. |
| 2 Please include additional information regarding the survey or questionnaire used in the study and ensure that you have provided sufficient details that others could replicate the analyses. For instance, if you developed a questionnaire as part of this study and it is not under a copyright more restrictive than CC-BY, please include a copy, in both the original language and English, as Supporting Information, or include a citation if it has been published previously. | We would like to thank you for this good suggestion. We have provided the survey or questionnaire used in the study (please see the supplementary file). |
| 3 Please provide further details on sample size and power calculations. | Thanks for this feedback. We have now provided further details on sample size and power calculations in revised version of manuscript. Please see, L No 123 to 129. |
| 4. In statistical methods, please clarify whether you corrected for multiple comparisons. | Thank you for the comments and suggestions. Multiple comparisons tests (MCTs) are performed several times on the mean of experimental conditions. However, in this study we did not do any multiple comparisons test. |
| 5. Please include your tables as part of your main manuscript and remove the individual files. Please note that supplementary tables (should remain/ be uploaded) as separate "supporting information" files | We would like to thank you for your constructive suggestion. We have now included the tables as part of main manuscript and removed the individual files in revised main manuscript. |
| 6. We note that you have indicated that data from this study are available upon request. PLOS only allows data to be available upon request if there are legal or ethical restrictions on sharing data publicly | We would like to thank you for your suggestion. We will provide the dataset which was used in this study. |
| A. If there are ethical or legal restrictions on sharing a de-identified data set, please explain them in detail (e.g., data contain potentially identifying or sensitive patient information) and who has imposed them (e.g., an ethics committee). Please also provide contact information for a data access committee, ethics committee, or other institutional body to which data requests may be sent | We will provide the dataset which was used in this study.  In addition, we have now added a section on ‘Ethical approval and respondents’ consent’. Please see L No 174 to 179. |
| B. If there are no restrictions, please upload the minimal anonymized data set necessary to replicate your study findings as either Supporting Information files or to a stable, public repository and provide us with the relevant URLs, DOIs, or accession numbers. Please see http://www.bmj.com/content/340/bmj.c181.long for guidelines on how to de-identify and prepare clinical data for publication | We would like to thank you for your suggestion. We will provide the dataset which was used in this study. |

| **Reviewers' comments** | **Answer** |
| --- | --- |
| **Reviewer #1** |  |
| 1. The content of the MS doesn't look sound enough to be a research article, it is a collection of some information and put it in one place systematically. Only with this content it does not match with the research article category of the PLOS ONE | We humbly disagree with the reviewer’s notion about our manuscript. Because, this paper has all elements of a research article. We have conducted a primary survey using a structured questionnaire following proper protocol. A sound methodology is used to answer the research questions of this study. The results, discussions and conclusion were presented in an informative way. This paper has added new knowledge in this field of research. |
| **Reviewer #2** |  |
| 1. **Abstract:** |  |
| - Word limits excided as per Journal guidelines. Should be modified. | Thank you for your comment; we have now kept the word limits within journal requirement. |
| - Write name of district (L. No. 26) | We have added district name in the revised manuscript. Please see, L. No 26. |
| - L No. 32: 50-60 year age will be the higher age category. Author has advised to confirm the age category | Corrected in revised manuscript. Please see L No 31. |
| **2 Introduction:** |  |
| Author has advised to shorten this section and put some point in to the discussion section. | Thank you for your information, we have shortened this section and added some part in method section. Please see L No. 105 to 114**.** |
| **3. Method** |  |
| Author should specify the study duration (Date), instead of Mid- June 2020 to mid- August 2020 (L. No. 129-130) | Thank you for this comment, and we specify the study duration instead of Mid- June 2020 to mid- August 2020 in revised manuscript. Please see, L No 117. |
| Ethical statement should write in separate sub-section. | We have now added a section, ‘Ethical approval and respondents’ consent’ in the revised manuscript. Please see line 174 to 179. |
| **4.Results** |  |
| Author has advised to rewrite the results and specify the Table legends as according to the Journal guideline. | We regret that we did not understand what actually we are asked to do by this sentence “Author has advised to rewrite the results”. Probably we are asked to rewrite the results. But we have not provided any explanation of what should we target to rewrite the results. Anyway, we have made some changes.  We have now followed journal guidelines thoroughly including Tables guideline. |
| **5.Discussion**: |  |
| An abbreviation of Tuberculosis (TB) and anti- tuberculosis (anti-TB) were written anonymously by authors. Please correct. | We would like to thank the reviewer for this constructive suggestion. In the revised manuscript, an abbreviation of Tuberculosis (TB) and anti- tuberculosis (anti-TB) were concisely written throughout the paper. We use full name Tuberculosis, when it is mentioned for the first time, then we use TB afterward throughout the paper. |
| Authors should specify there limitation in present study. | Thank you for your suggestion, we updated our limitation of the study in revised manuscript. Please see, L No 322 to 326 |
| Conclusive statements should also modify to understand readers. | We feel that the conclusion of this study was clear enough for the readers, however, we have made a few modifications to make it further clearer. |
| Author has advised to use universal/ single system while writing the reference. | Thank you for this feedback, we have now accommodated this feedback in the revised manuscript. |
